# Characterization of the *Liriodendron Chinense MYB* Gene Family and Its Role in Abiotic Stress Response

**DOI:** 10.3389/fpls.2021.641280

**Published:** 2021-07-26

**Authors:** Weihuang Wu, Sheng Zhu, Liming Zhu, Dandan Wang, Yang Liu, Siqin Liu, Jiaji Zhang, Zhaodong Hao, Ye Lu, Tielong Cheng, Jisen Shi, Jinhui Chen

**Affiliations:** ^1^Key Laboratory of Forest Genetics and Biotechnology, Ministry of Education of China, Co-Innovation Center for the Sustainable Forestry in Southern China, Nanjing Forestry University, Nanjing, China; ^2^College of Biology and the Environment, Nanjing Forestry University, Nanjing, China

**Keywords:** *Liriodendron chinense*, genome-wide, transcription factor, *MYB* gene family, abiotic stress

## Abstract

*Liriodendron chinense* (*Lchi*) is a Magnoliaceae plant, which is a basic angiosperm left behind by the Pleistocene and mainly distributed in the south of the Yangtze River. *Liriodendron hybrids* has good wood properties and is widely used in furniture and in other fields. It is not clear if they can adapt to different environmental conditions, such as drought and high and low temperatures, and the molecular mechanisms for this adaptation are unknown. Among plant transcription factors (TFs), the *MYB* gene family is one of the largest and is often involved in stress or adversity response signaling, growth, and development. Therefore, studying the role of MYBTFs in regulating abiotic stress signaling, growth, and development in *Lchi* is helpful to promote afforestation in different environments. In our research, a genome-wide analysis of the *LchiMYB* gene family was performed, including the phylogenetic relationship tree, gene exon-intron structure, collinearity, and chromosomal position. According to the evolutionary tree, 190 *LchiMYBs* were divided into three main branches. *LchiMYBs* were evenly distributed across 19 chromosomes, with their collinearity, suggesting that segment duplication events may have contributed to *LchiMYB* gene expansion. Transcriptomes from eight tissues, 11 stages of somatic embryogenesis, and leaves after cold, heat, and drought stress were used to analyze the function of the *MYB* gene family. The results of tissue expression analysis showed that most *LchiMYB* genes regulated bark, leaf, bud, sepal, stigma, and stamen development, as well as the four important stages (ES3, ES4, ES9, and PL) of somatic embryogenesis. More than 60 *LchiMYBs* responded to heat, cold, and drought stress; some of which underwent gene duplication during evolution. *LchiMYB3* was highly expressed under all three forms of stress, while *LchiMYB121* was strongly induced by both cold and heat stress. Eight genes with different expression patterns were selected and verified by quantitative real-time PCR (qRT-PCR) experiments. The results suggested that these *LchiMYBs* may regulate *Lchi* growth development and resistance to abiotic stress. This study shows the cross-regulatory function of *LchiMYBs* in the growth and development, asexual reproduction, and abiotic resistance of *Lchi*. This information will prove pivotal to directing further studies on the biological function of *Lchi* MYBTFs in genetic improvement and abiotic stress response.

## Introduction

Plants respond to different environmental conditions through a series of physiological and metabolic processes (Chen et al., 2017; Li J. et al., 2019). At every stage of plant growth and development, there are many genes involved in regulating its biological processes (Dubos et al., 2010). Transcription factors (TFs), which bind to upstream sequences of genes, regulate gene expression and play a vital role in regulating biological processes (Ambawat et al., 2013; Li Y. et al., 2020). Generally, TFs are composed of at least four domains, including a transactivation domain, a DNA-binding domain, a nuclear localization signal, and oligomerization sites (Stracke et al., 2001; Dubos et al., 2010). According to the types of conserved domains, TFs can be divided into many families. The *MYB* gene family exists in all eukaryotes, especially in the plant kingdom. It is one of the largest families and has functional diversity (Dubos et al., 2010; Ambawat et al., 2013).

The highly conservative MYB domain of MYB family members comprises 1–4 neighboring incomplete tandem repeats. Each repeat unit consists of an average of 50–53 amino acid (aa) residues and forms three α-helices. The second and third repeats have a helix-turn-helix (HTH) secondary structure; these repeats bind in the upstream regions of the target genes (Stracke et al., 2001; Chen et al., 2017; Wei et al., 2020). However, MYB TFs possess many regulatory roles, as the more variable C-terminus of MYB proteins (Zhang et al., 2020). According to the number of neighboring MYB repeats, MYBs are divided into four capital subgroups: MYB-related (1R-MYB), R2R3-MYB (2R-MYB), R1R2R3-MYB (3R-MYB), and 4R-like MYB (4R-MYB). In the plant kingdom, *2R-MYBs* are one of the maximal and most common MYBs and are considered to have two main evolutionary modes: the R1R2R3-MYB domain through the lack of an R1 repeat and the duplication of an R1 repeat in the R1-MYB domain (Dubos et al., 2010; Ambawat et al., 2013).

Since the discovery of the first plant *MYB* gene C1, the identification and functional characteristics of the plant *MYB* gene family have been widely reported (Dubos et al., 2010). A total of 519 TFs expressed in *Arabidopsis* are involved in regulating somatic embryogenesis (Gliwicka et al., 2013). Many TFs, such as LEAFY COTYLEDON (LEC), WUSCHEL (WUS), BABY BOOM (BBM), Arabinogalactan-proteins (AGPs), CLAVATA (CLV), and APETALA 2 (AP2), are involved in regulating the somatic embryogenesis process (Méndez-Hernández et al., 2019). WRKY, MYB, AIL, and bHLH regulate the later phase of somatic embryogenesis in rubber (Wang et al., 2021). In different tissues, *AtMYB113* and *AtMYB114* are expressed in vegetative tissues and regulate anthocyanin biosynthesis (Dubos et al., 2010). Eight *BdMYB* genes were expressed specifically in leaves of *Brachypodium distachyon* (Chen et al., 2017). Some *R2R3-MYB* genes had the highest expression in the pericarp of *capsicum annuum* L. (Wang J. et al., 2020).

Recently, numerous studies have focused on the response of the *MYB* family members to plant stress. In *Arabidopsis*, *AtMYB15* responds to cold stress and has a negative regulatory role in the CBF signaling process (Shen et al., 2019). In rice, *OsMYB2* responds to salt, cold, and drought stresses (Jiang et al., 2004; Katiyar et al., 2012). The *TaMYB sdu1* gene in wheat (*Triticum aestivum L.*) has an important role in responding to salt and drought stresses (Rahaie et al., 2010). Overexpression of TaMYB34 in transgenic tobacco can enhance tolerance to drought, high temperature and high salt stress (Wei et al., 2020). In poplar, transgenic tobacco overexpressing PsnMYB108 gene has stronger salt tolerance than wild-type tobacco (Zhao et al., 2020).

The *Liriodendron* genus belongs to the Magnolia family. Other than the Pleistocene, there exist only two species in this genus, i.e., the East Asian [*L. chinense (Hemsley) Sargent*] species and the North-East American species (*L. tulipifera Linn*) (Chen et al., 2019). Two separate lineages of *Liriodendron chinense* (*Lchi*) are found on the Chinese mainland, comprising the eastern and western groups (Yang et al., 2016; Chen et al., 2019). *Liriodendron hybrids* is intraspecific hybrids crossed by *L. chinense* and *L. tulipfera*, which was a valuable ornamental tree species in both private and public spaces because of its fast growth, good wood properties, and wide use in furniture making and other fields (Hu et al., 2017; Li et al., 2021).

Complete genome sequencing of *Lchi* showed that magnolia is an ancient angiosperm, which is present before monocotyledon and dicotyledon differentiation (Chen et al., 2019). Therefore, it could provide valuable information on how plants from such an ancient phylogenetic branch cope with abiotic stress. *Lchi* is mainly distributed in the south of the Yangtze River (Chen et al., 2019); there are few studies on its ability to adapt to different environments, such as colder northern China, warmer southern China, or arid northwestern China. The *MYB* gene family has important functions in the growth, development, and response to abiotic stress, but studies on the whole genome of *Lchi* have rarely been reported.

In this research, the *MYB* family was systematically analyzed in the whole genome of *Lchi*, and 190 *LchiMYBs* were divided into three main groups (1R-MYB, 2R-MYB, and 3R-MYB). An overall analysis, including the determination of exon-intron structure, a gene duplication event, chromosome location, phylogenetic analysis, and synteny analysis, was performed. Furthermore, the specifically expressed *MYB* gene family members were identified through transcriptome analysis of vegetative and reproductive organs, somatic embryogenesis, and abiotic stressors of high and low temperatures and drought in *Lchi.* The results suggest that *LchiMYBs* have a vital role in the growth and development of *Lchi* and its adaptability to adversity.

This study provides insights into the regulatory function of *LchiMYB* in the growth and development, asexual reproduction, and abiotic resistance of *Lchi*, which provide a theoretical basis for the genetic improvement of *Lchi*, thereby increasing forest yield and promoting afforestation.

## Materials and Methods

### Datasets and Sequence Retrieval

The complete genome, transcript/protein sequences, and the genome feature file of *Lchi* were downloaded from https://www.ncbi.nlm.nih.gov/assembly/GCA_003013855.2. All MYB proteins of *Arabidopsis thaliana* were obtained from the TAIR database^1^. An MYB Hidden Markov profile (PF00249) was retrieved from the Pfam website^2^, and the MYB-domain-containing protein sequences in the *Lchi* genome were identified, using HMMER (v.3.0.1b, Cambridge University Press, England, United Kingdom) (with an *E*-value < 1E-5 and using this profile as a query. As a result, all candidate *LchiMYBs* were confirmed, using Pfam and the conservative Domains Database (CDD^3^) to validate the core domain sequences.

### Sequence Analysis

The gene exon-intron structural feature of each *LchiMYB* was acquired from the genomic feature file (GFF/GTF) and displayed, using the GSDS website the R2R3 domain “seqlogos” map was made through the WebLogo (http://weblogo.berkeley.edu/)^4^, while the chromosome location and microsynteny of *LchiMYBs* were visualized, using the R software “Circlize”^5^ (Gu et al., 2014)^5^. The presumptive orthologous gene pairs (E-value cutoff < 1E-20, identity > 70%) were verified by the Python software “MCScanx”^6^ and then used for comparative synteny analysis among two model plants (Wang et al., 2012). The *cis*-acting elements of *LchiMYB* upstream regions were predicted by PlantCARE^7^. Ka/Ks values were assigned to *LchiMYB*s, using the Ka/Ks_calculator (Nekrutenko et al., 2002). The basic properties of LchiMYB proteins, including protein length, isoelectric point (pI), and molecular weight (MW), were analyzed, using the ExPasy website^8^.

### Phylogenetic Analysis

Multiple sequence alignment (MSA) of *Lchi* and *A. thaliana* MYB-domain-containing proteins was done, using MUSCLE (v3.8.31), which was set to default parameters. The trimmed MSA file was used to construct the MYB phylogenetic tree, which was generated with trimAl (v1.4), set to the “automated1” mode (Capella-Gutierrez et al., 2009). The BEAST (v2.6.2) was used to perform MYB phylogenic analysis under the following settings: the maximum likelihood method, the substitution model was Dayhoff, priors set to the Yule Model, the birth rate was 1.0, the mcmc chain length was 10,000,000, bootstrapping set to 1,000, and the TreeAnnotator program set the posterior probability limit to 1.0 with a burn in percentage of 90 (Drummond et al., 2012). Then, the R software “ggtree” was used to visualize the phylogenic tree^9^ (Yu et al., 2018; Yu, 2020).

### Somatic Embryogenesis Transcriptome of *Liriodendron hybrids*

Embryogenic callus was induced from the embryo of seeds of a *Liriodendron hybrids*, and successive stages of somatic embryogenesis were used for transcriptome sequencing. The data have not yet been published. The 11 stages are as follows: proembryogenic mass cultured for 20 days for embryogenic callus (PEMs), then liquid suspension cultured for 10 days (ES1), single cell cultured for 2 days (ES2), induced by ABA for 1 day (ES3) and 3 days (ES4), a 7-day globular embryo (ES5), a 13-day heart-shaped embryo (ES6), a 19-day torpedo embryo (ES7), a 25-day immature cotyledon embryo (ES8), a 31-day mature cotyledon embryo (ES9), and a 37-day plantlet (PL). The Fragments Per Kilobase of exon model per million mapped fragments (FPKM) of transcriptome data is shown in the Supplementary Table 1.

### Various Tissue Transcriptomes of *Lchi*

The transcriptomes of various tissue types, namely, the phloem, stigma, xylem, bud, stamen, leaf, bark, and sepal, were used. The FPKM data of *LchiMYBs* are shown in the Supplementary Table 2. These transcriptome data have not yet been published.

### Plant Materials, RNA Extraction, qRT-PCR Analysis, and Transcriptome Sequencing Analysis of Abiotic Stress in *Liriodendron hybrids*

*Liriodendron hybrids* seedlings generated through somatic embryogenesis were used as the starting material throughout this study. Before any experiments were performed, plantlets were taken out of the culture medium vessel and acclimatized in a greenhouse for 2 weeks (22°C, long-time photoperiod of 16-h light and 8-h darkness, and 75% relative humidity). For three abiotic adversity treatments, plants were transferred to a growth incubator (long-time photoperiod of 16-h light and 8-h darkness, and 75% relative humidity). To simulate cold, heat, and drought stress, plantlets were subjected to 4°C, 40°C, or 15% PEG 6000 treatment, respectively, for 1 h, 3 h, 6 h, 12 h, 1 day, and 3 days. Each treatment consisted of five replicates for each sampling time. All experimental leaf tissue samples were frozen in liquid nitrogen immediately and then conservatively at −80°C for RNA extraction by RNA-seq analysis and quantitative real-time PCR (qRT-PCR) experiments.

We used the FastPure Total RNA Isolation Kit (Vazyme, Nanjing, China) (RC401) to isolate total RNA in leaves. Then, agarose gel electrophoresis and a Nanodrop ND-1000 spectrophotometer were used to validate the quality of the extracted RNA. A HiScript^®^ III 1st Strand cDNA Synthesis Kit (Vazyme, Nanjing, China) was used to synthesize cDNA with the extracted RNA as a template.

*Lchi* leaf tissue transcriptome after the three stressors was determined with RNA-seq on biological triplicates by using the extracted RNA samples and then mapping the clean reads, using our genome sequence as a reference. An RNA-seq analysis software pipeline was employed, consisting of hisat2 (v2.0.5) to map clean reads to the genomic sequence and Kallisto (v0.46.1) to count transcripts per million (TPM) values of individual genes. The TPM values of all mapped *LchiMYBs* (35269) are shown in Supplementary Table 3. DESeq2 (v 1.16.1) used analysis of variance (ANOVA; *P*.adjust < 0.05, | Log2FC| > 1) to determine the genes that were differentially expressed. TPM was used to calculate the transcript abundance of *LchiMYB* genes. Expression heatmaps were created, using the R package “pheatmap” (v1.0.12), with the log 2 (TPM + 1) values. The transcriptome data of the present study were archived and can also be obtained on NCBI (cold and heat: PRJNA679089; drought: PRJNA679101).

Primers of eight *MYB* genes selected by RNA-seq results with different expression patterns were used for quantifying the expression profile and were designed through the primer 3 website^10^. The RNA samples used for quantitative RT-PCR (qRT-PCR) were the same as those for RNA-seq. qRT-PCR was implemented with a Roche Lightcyler^®^ 480II instrument, using 2x AceQ^®^ qPCR SYBR^®^ Green Master Mix (Without ROX) (Vazyme, Nanjing, China). The composition of the PCR mix was as follows: 10 μl of 2x AceQ^®^ qPCR SYBR^®^ Green Master Mix (without ROX), 0.4 μl of each primer, 1 μl of a cDNA template (10 ng/μl), and 8.2 μl of ddH_2_O for a final volume of 20 μl. The internal reference gene chosen for housekeeping was the *Lchi 18S* gene. The reaction was implemented as follows: 95°C for 30 s and then 45 cycles of 95°C for 10 s and 60°C for 30 s. All experiments were run in 96-well plates. Each experiment was carried out in biological triplicates, as well as three technical replicates. All data generated from qRT-PCR were calculated by the 2^–ΔΔCT^ formula. The melt curve of primers is shown in the Supplementary Figure 1, and sequences are also shown in Supplementary Table 4.

Sequence analysis was used to obtain the number of *LchiMYB* family members, physical and chemical properties of the protein, replication events, and promoter functions of regulating growth, development, and abiotic stress response. Phylogenetic analysis further analyzed the grouping specificity of *MYB* genes and explored their expression patterns in the growth, development, and response to abiotic stress according to the grouping specific functions of these genes. Therefore, abiotic stress experiments and RNA-seq of tissue and somatic embryogenesis were carried out. Finally, eight genes with different expression patterns for abiotic stress were selected for qRT-PCR verification.

## Results

### Identification of *LchiMYBs* and Corresponding Protein Sequence Features

To investigate the *MYB* genes within the *Liriodendron chinense* (*Lchi*) genome, the MYB hidden Markov model (PF00249) was used as a query in hmmsearch searches against the *Lchi* genome-wide protein sequences. Many MYB protein sequences (>250 candidates) that had MYB or MYB-like repeats were obtained. In Pfam, these sequences were further used to verify the completeness of the MYB domain in the identified sequences, discarding those with or without incomplete MYB domains. Finally, a total of 190 non-redundant LchiMYB proteins were obtained, including 76 (40%) R2R3-MYBs (2R-MYBs), 109 (57.37%) MYB-related (1R-MYBs) proteins, and 5 (2.63%) R1R2R3-MYBs (3R-MYBs). Together, they accounted for 0.5387% of all annotated *Lchi* genes (35,269), which is a larger fraction than that in rice (0.3934%), yet smaller than that in *Arabidopsis* (0.5964%), which is of the same family. No 4R-MYB proteins were identified, possibly due to the incompleteness of the *Lchi* genome. The 1R-MYB proteins accounted for more than half of the total number of MYB proteins, thus constituting the largest group of *MYB* genes. According to the results of this study, most *MYB* genes resided within the R2R3-MYB subgroup, yet *Lchi* 2R-MYBs represent less than half of the total number of MYB proteins, which contrasts with previous studies on rice and *Arabidopsis*. Perhaps, this is one aspect that differentiates basic angiosperms, such as *Lchi*, from higher angiosperms.

The predicted *LchiMYB* genes were numbered from *LchiMYB1* to *LchiMYB165* based on their coordinate order on *Lchi* chromosomes, and *LchiMYB166*–*LchiMYB190* were numbered based on their order on sequenced scaffolds. The characteristics of *LchiMYB* genes, including their chromosome position, protein MW, amino acid length, and pI, were further analyzed (Supplementary Table 5). The smallest protein was LchiMYB180 at 75 amino acids (aa) in length, and the largest was LchiMYB51 at 1,374 aa, while the MW ranged from 8.67 (LchiMYB100) to 149.2 (LchiMYB158) kDa, and the pI ranged from 4.34 (LchiMYB150) to 10.61 (LchiMYB48).

**FIGURE 1:**
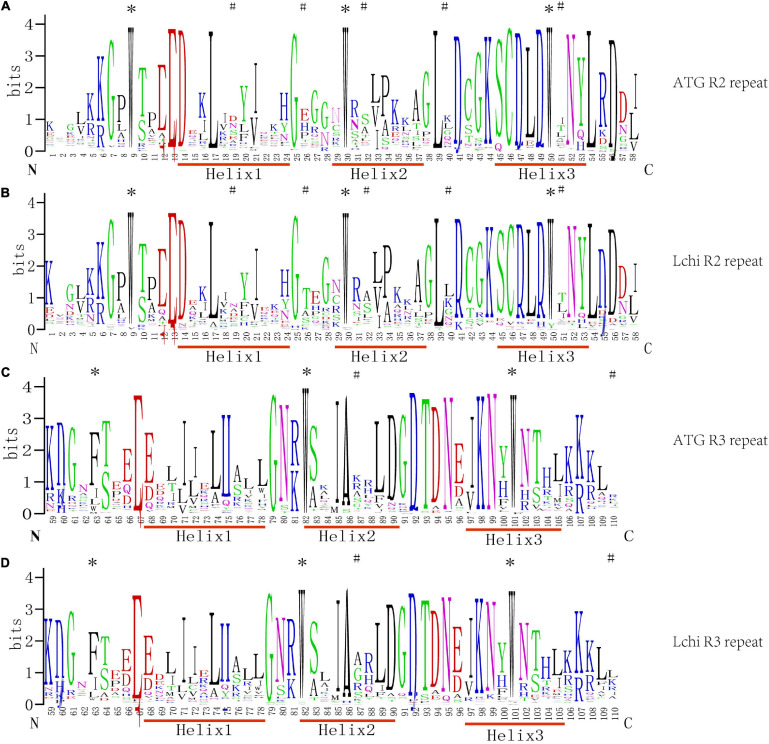
Sequence logos of the R2 and R3MYB repeats from *Liriodendron chinense* (*Lchi*) and *Arabidopsis*. Multiple sequence alignment analysis of 76 *Lchi* and 126 *Arabidopsis* R2R3-MYB domains was used in ClustalX2.1. Panels **(A,B)** were the R2 repeats, and panels **(C,D)** were the R3 repeats in *Arabidopsis* and *Lchi*. N and C represent the N-terminal and C-terminal, respectively, and the bit score shows the conservative information for all positions in the sequence; the higher the value, the higher the conservation, and the different colors were generated by the website randomly. The asterisks represent the highly conserved tryptophan (W) and phenylalanine (F) residues, and the ^#^ represents the different patterns of positions between *Lchi* and *Arabidopsis*.

To detect the sequence characteristics of MYB and the base frequency of each amino acid site, 76 homologous R2R3-MYB sequences were analyzed by MSA, and the seqlog map of sequence frequency was obtained (Figure 1).

In general, R2R3-MYB domains have, on average, approximately 108 amino acids between each MYB protein (Liu et al., 2020; Pucker et al., 2020). Outside the DNA-binding domain, however, the length and the composition of amino acids vary greatly. The LchiMYB proteins show a similar pattern, confirming the conserved nature of the MYB domain (Sun et al., 2019; Wang et al., 2019).

As shown in Figure 1, the amino acid distribution frequencies within the LchiMYB domain repeat closely and resemble those of many previously reported plant species; for example, grapes, Arabidopsis, poplars, and pears. However, compared with other plants, the R2 and R3 MYB repeats of LchiMYB proteins contain some unique amino acids, including several highly conserved and evenly distributed tryptophan (W) residues, which are usually used as a label of MYB proteins in plants. According to previous reports, the first conserved amino acid of the R3 repeat is often replaced by Phe (F) or Ile (I). However, Trp at position 63 is often replaced by the amino acid Leu (L) in LchiMYB proteins. In addition to the highly conserved tryptophan residues, the amino acids in other regions of the LchiMYB protein are also conserved, such as Gly-25 (G), Cys-46 (C), and Arg-49 (R) in the R2 repeat, Leu-54 (L) in the linker region, and Glu-66 (E), Thr-87 (T), and Arg-94 (R) in the R3 repeat. In other species, such conserved amino acids are also found, which are mainly related to the third helix and the second turn motif (Millard et al., 2019; Zhou et al., 2019). Additionally, some residues in *Lchi* were distinct from *Arabidopsis*, such as the patterns at positions 19, 26, 32, 40, and 51 of R2 and 87 and 110 of R3, which show the divergence of the MYB domain.

These results suggest that the diverse physical and chemical properties of LchiMYB, fewer R2R3 members, and the R2R3-MYB domain were very conservative in plants. Therefore, it is necessary to further explore the characteristics of the *LchiMYB* gene family.

### Chromosome Distribution, Duplication Event, and Collinearity Analysis of *LchiMYBs*

**FIGURE 2:**
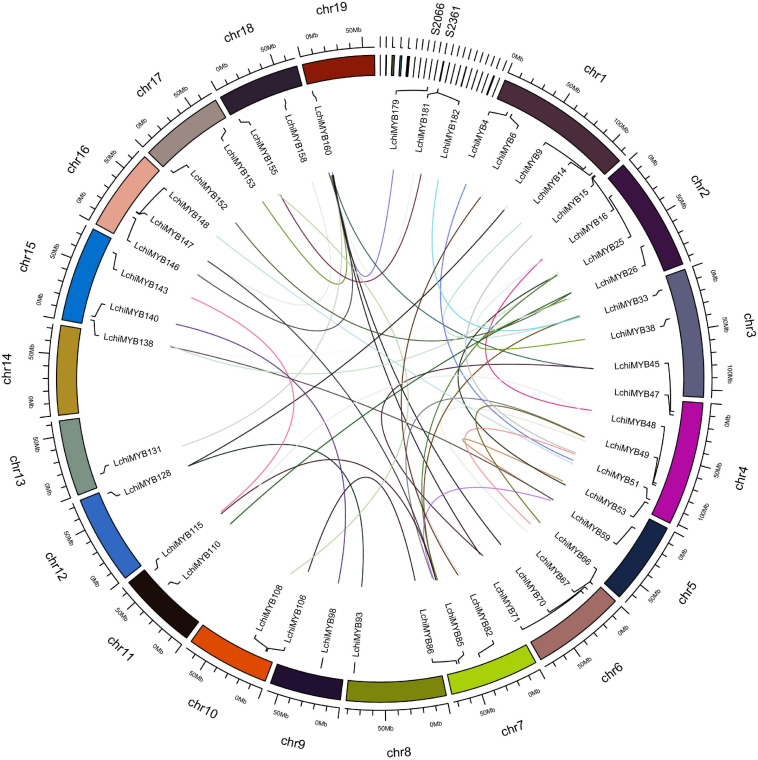
Chromosomal distribution and syntenic relationships of *LchiMYBs*. The colored curve lines represent duplicated *MYB* gene pairs. *MYB* family genes are located on all chromosomes and several scaffolds. Only duplicated gene pairs are shown; all 19 chromosomes are labeled, and only two scaffolds are labeled.

We continued characterizing the *LchiMYB* genes by charting their chromosomal positions. This analysis showed that the 190 *LchiMYB* genes are unevenly distributed across the 19 *Lchi* chromosomes, with 25 *LchiMYB* genes anchored to 21 unassembled scaffolds (Supplementary Tables 6, 7 and Figure 2).

Chromosome 1 contains the most *LchiMYB* genes (17), followed by chromosome 4 (16). In contrast, chromosomes 14 and 17 only had three *LchiMYB* genes. There was no significant correlation between the number of *LchiMYBs* on a chromosome and the length of the chromosome.

The expansion of this gene family was mainly caused by gene replication events, including tandem and segment repeats. In this study, the collinearity of the MYB gene family was analyzed in the genome of *Lchi*, and gene replication events were explored. The results are shown in Figure 2. All chromosomes with an *MYB* gene are shown, and only duplicated genes are labeled on the chromosomes. A total of 40 segmental duplication events and 46 *MYB* genes were identified. Some *LchiMYB* genes had multiple replication events, such as *LchiMYB48*, *LchiMYB110*, and *LchiMYB26*. The *LchiMYB* genes are mainly distributed in synteny blocks on 18 chromosomes and two scaffolds. Generally, two or more genes arranged within 20 kb are defined as a gene cluster (Zhou et al., 2015). In this study, we identified two *LchiMYB* genes (*LchiMYB20* and *LchiMYB21*) in a gene cluster. Ka/Ks (a non-synonymous/synonymous substitution ratio) values were calculated for all *MYB* gene pairs with segment duplication events (Supplementary Tables 8, 9). Twenty-two gene pairs had ka/ks values more than 1, and the ka/ks values of 18 gene pairs were less than 1, indicating that the *LchiMYB* gene was mainly subjected to positive selection pressure during evolution (Jia et al., 2003; Katiyar et al., 2012; Sun et al., 2019).

**FIGURE 3:**
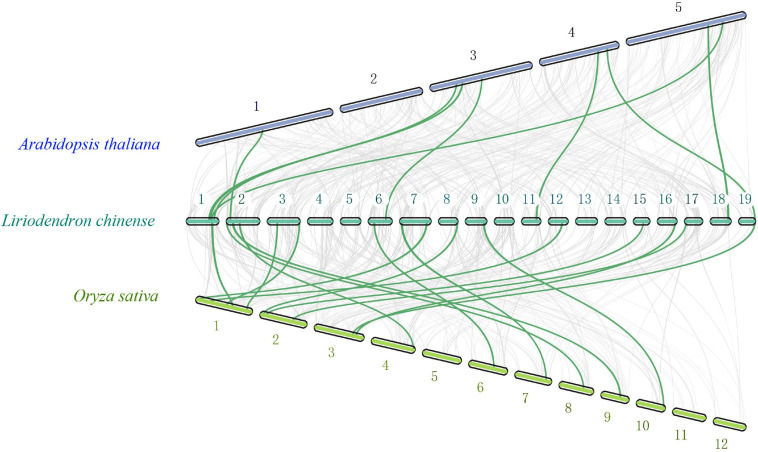
Collinearity analyses of *MYB* genes among *Lchi*, *Oryza sativa*, and *Arabidopsi*s. The genomic linear blocks of *Lchi*, *Oryza sativa*, and *Arabidopsis* are represented by gray lines, while the syntenic *MYB* gene pairs are highlighted by the green lines. The number on the chromosome represents the chromosome ordinal number (Wang et al., 2012).

To further study the evolutionary status of the *LchiMYB* gene family, a genome-wide comparative syntenic analysis was conducted, using three species, namely, *Lchi*, *Arabidopsis thaliana*, and *Oryza sativa* (Katiyar et al., 2012; Ambawat et al., 2013), which belong to the basic angiosperms, dicotyledonous plants, and monocotyledonous plants, respectively (Figure 3).

There were eight syntenic gene pairs between *A. thaliana* and *Lchi*, and 16 gene pairs between *O. sativa* and *Lchi*, which indicated that the evolutionary status of MYB in *Lchi* might be closer to that of *O. sativa* and consistent with that of its genome (Katiyar et al., 2012). The formation of these specific syntenic gene pairs may have resulted from the evolutionary status of magnolia species, in which basal angiosperms were formed before the separation of monocotyledons and dicotyledons (Chen et al., 2019). These results demonstrated that *LchiMYBs* are randomly distributed on chromosomes and mainly suffer from active selection. In the evolutionary process, it may have formed in the basal angiosperm stage. Thus, their gene structure, classification, and functional features need to be further explored.

### Phylogeny Classification, Gene Exon–Intron Structure, and *Cis* Elements of *LchiMYBs*

**FIGURE 4:**
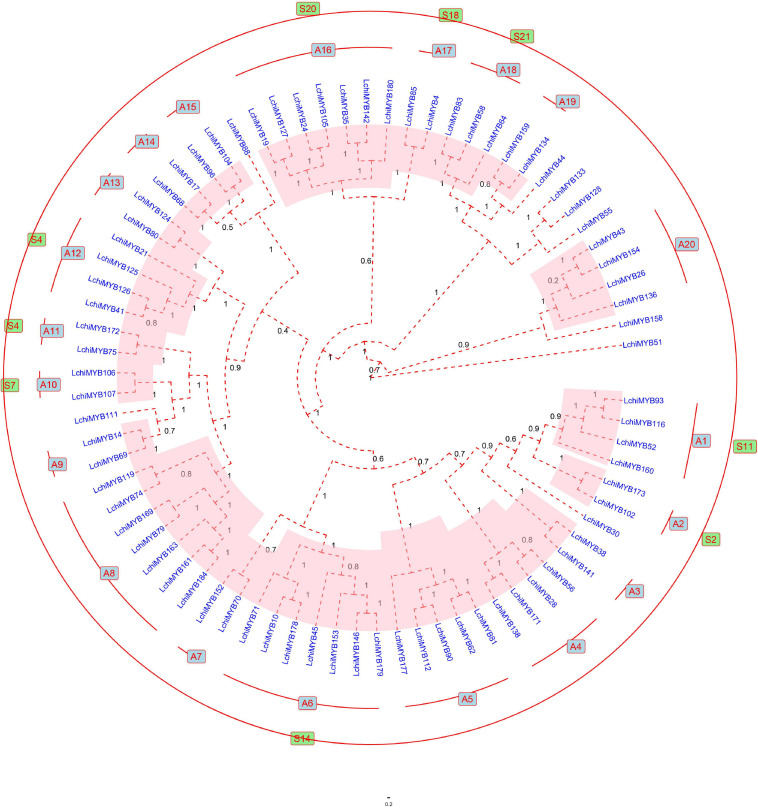
The Bayesian tree showing the phylogenetic relationships between 76 *Lchi* R2R3-MYB proteins. The protein sequences are divided into 20 main groups, each marked with a subgroup number (e.g., A1). The groups homologous to *Arabidopsis* are labeled outside the evolutionary tree and are indicated by the color green. The bootstrap values are supported by 1,000 replications and are shown beside the branches. Only 11 proteins did not cluster into any group.

To further explore the classification, gene exon-intron structure, and function of *LchiMYBs*, a Bayesian phylogenetic tree was constructed from the 190 *LchiMYBs*, which were classified into three groups (Supplementary Figure 2 and Supplementary Table 10). As *R2R3-MYBs* are very important in plants and have been reported in many kinds of research, 76 full-length R2R3-MYBs from *Lchi* were used to construct the Bayesian phylogenetic tree to classify *LchiMYB* genes (Liu et al., 2017). Based on the phylogenetic tree classification of *Arabidopsis* R2R3-MYB proteins, *Lchi* was divided into 20 subgroups (designated A1–A20) according to the similarity of protein sequences and the branches of the phylogenetic tree (Figure 4).

The R2R3-MYB Bayesian phylogenetic tree of *Lchi* (76) and *Arabidopsis* (125) are presented in Supplementary Figure 3 and Supplementary Table 11. Most large subfamilies (A6, A8, and A15) have high bootstrap values, which are consistent with previous reports. However, certain smaller subfamilies (A3, A7, and A9) have no homologous groups with *A. thaliana* (Li and Lu, 2014; Zhao et al., 2014), and seven LchiMYB proteins were not grouped.

**FIGURE 5:**
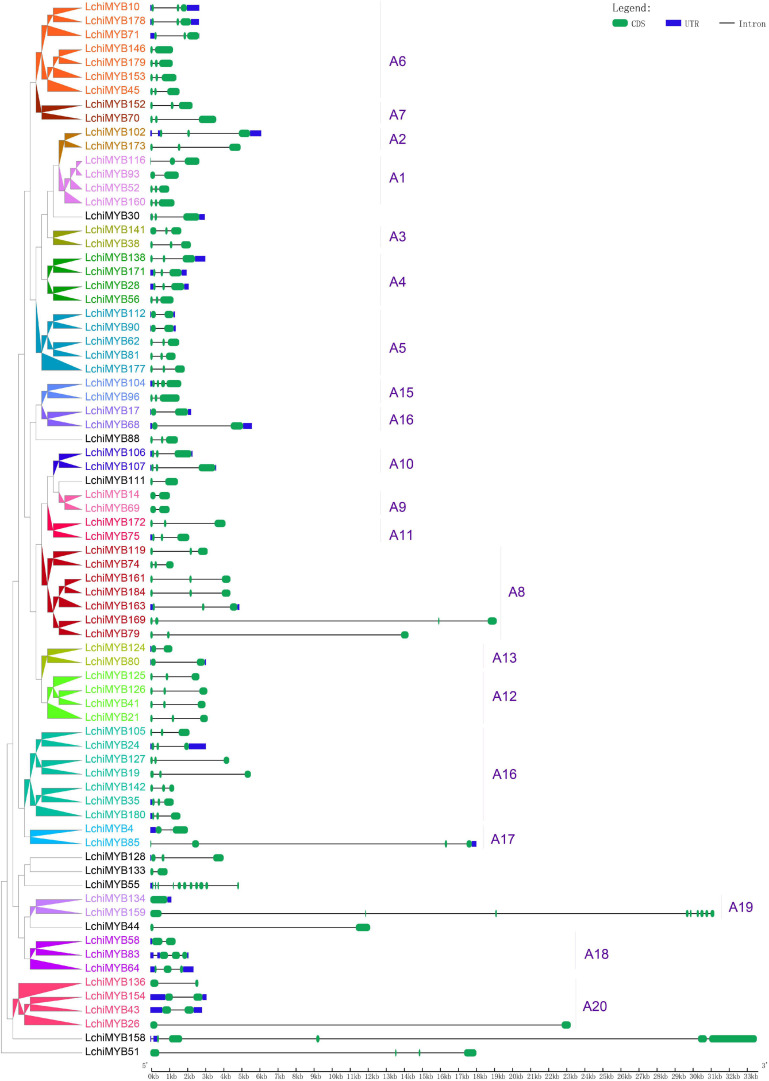
*Lchi* MYB gene exon-intron structures. Blue rectangles indicate untranslated 5′– and 3′– regions; green rectangles indicate exons; black straight lines represent introns; the tick marks represent the length of the gene. The corresponding groups were labeled (e.g., A1), with different colors on the branches and gene labels.

Following phylogenetic analysis, exon-intron structure analysis was performed for the 76 full-length *Lchi R2R3-MYB* genes. Most *Lchi R2R3-MYB* genes contained introns, except one gene (*LchiMYB134*) from the subgroup A19 (Supplementary Table 12 and Figure 5).

The number of introns in the *MYB* gene ranged from 0 to 3, except for three genes (*LchiMYB158*, *LchiMYB159*, and *LchiMYB55*), which had 4, 9, and 10, respectively. More than half (45/76) of the *LchiMYB* genes had three exons; the results of these gene structures are consistent with those of the same group with similar gene structures in the evolutionary tree, such as A2, A3, and A10. However, there are two exceptions: subgroups A17 and A19, which contained different members of introns. Overall, similar gene structures and phylogenetic tree groupings strongly support the reliability of our grouping classifications (Yanhui et al., 2006; Wilkins et al., 2009).

**FIGURE 6:**
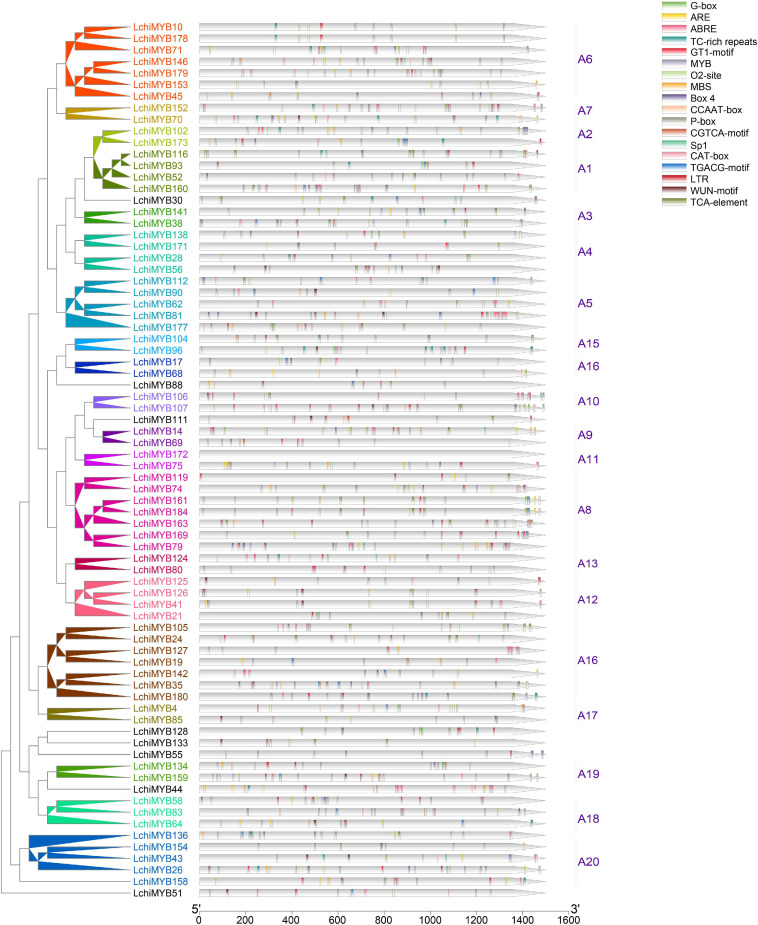
*LchiMYB cis*-acting elements. Displayed is an unrooted phylogenetic tree constructed, using Beast (v2.6.2) set to the Bayesian method. The legend contains 18 *cis*-acting elements identified within the 1.5-kb long *LchiMYB* promoter sequences; the tick marks represent the length of the promoter sequences. Different *cis*-acting elements are color coded. The corresponding groups were labeled (e.g., A1) with different colors on the branches and gene labels.

To further understand *LchiMYB* gene function, the PlantCARE website (see footnote 7) was used to identify known *cis* elements within the 1.5-kb upstream region of each gene (Figure 6).

Two types of *cis* elements were detected: one related to plant growth and development, and the other related to stress responses (Supplementary Table 13) (Srideepthi et al., 2020). *Cis* elements related to growth and development include the MYB binding site (MYB), light responsive (Box-4, G-box, GT1-motif, Sp1), an MYBHv1 binding site (CCAAT-box), meristem-specific activation (O2-site), and meristem expression (CAT-box). *Cis* elements related to abiotic stress include anaerobic induction (ARE), methyl jasmonate (MeJA) response (TGACG-motif and CGTCA-motif), ABA-responsive element (ABRE), salicylic acid (SA) response (a TCA element), gibberellin responsive (P-box), defense and stress response (TC rich), drought response (MBS), low-temperature response (LTR), and wound-responsive (WUN-motif) (Chen et al., 2017). These results showed that *LchiMYB* genes regulate abiotic stress signaling, and that they potentially regulate each other during growth and development. Therefore, it is possible that, under abiotic stress conditions, *LchiMYBs* may change their expression patterns, and they also play a regulatory role in different growth and developmental stages.

### Expression Profiling of *LchiMYB* Genes in Vegetative and Reproductive Organs of *Lchi*

**FIGURE 7:**
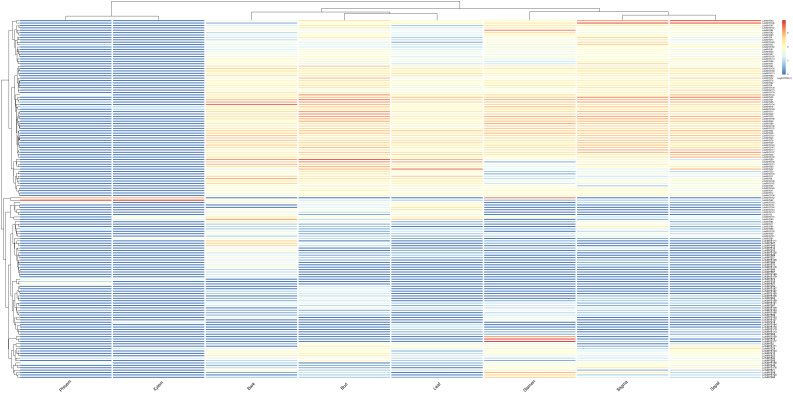
The heatmap of FPKM values in eight different tissues. The phloem, xylem, stigma, bark, bud, leaf, stamen, stigma, and sepal represent the eight different tissues. The transcript abundance level was normalized and hierarchically clustered by using the log 2 (FPKM + 1) comparison among genes per tissue and different tissues. Colored blocks show decreased (blue) or increased (red) transcript accumulation at different stages. The color legend indicates the fold change expression of *LchiMYBs*, changing from low (blue) to high (red) expression. The heatmap was generated, using the R package “pheatmap” (v 1.0.12).

To explore the spatial expression pattern of *LchiMYBs*, the expression profiles of different tissues were studied to determine the function of *MYBs* based on previous reports. *LchiMYBs* regulate development in different tissues and perform differential expression profiling in *Lchi* (Chen et al., 2017). As shown in Figure 7, a total of 150 (78.95%) genes were differentially expressed in eight tissues, namely bark, phloem, xylem, leaf, bud, sepal, stigma, and stamen.

Many *LchiMYBs* were highly expressed in the bark, leaf, and stamen, followed by the bud, stigma, and sepal. Interestingly, only a few genes were expressed in the phloem and xylem tissues; perhaps, *LchiMYBs* have no obvious functions in these two tissues. For example, *LchiMYB152* and –*82* were only expressed highly in the phloem, xylem, and stamen, but not in other tissues. *LchiMYB118* and –*90* were highly expressed in the bark and leaf and nearly not expressed in other tissues. *LchiMYB70*, –*177*, and –*2* were highly expressed in the stamen. This suggests that specific tissues are regulated by different genes.

Moreover, some *LchiMYBs* showed multi-tissue regulatory functions, like *LchiMYB124*, –*26*, –*91*, and –*98*, which were highly expressed in the stigma, bud, stamen, leaf, bark, and sepal but not in the phloem and xylem. Some genes were less expressed in all tissues, such as *LchiMYB54*, –*187*, –*167*, and –*190*, which were only less expressed in the bud and bark. These results showed that *LchiMYBs* have diversified regulation modes in different tissues, and there are tissue-specific genes.

### Expression Profiling of *LchiMYB* Genes During 11 Successive Stages of Somatic Embryogenesis in *Liriodendron hybrids*

Embryogenic callus was induced by embryos of seeds gained from a *Liriodendron* hybrid, which was the offspring produced by artificial pollination (*L. chinense* × *L. tulipifera*), and the 11 successive stages of somatic embryogenesis were sequenced, namely PEMs, ES1, ES2, ES3, ES4, ES5, ES6, (ES7), (ES8), (ES9), and (PL). According to previous reports, the *MYB* gene has a regulatory function in somatic embryogenesis (Wang F. X. et al., 2020).

**FIGURE 8:**
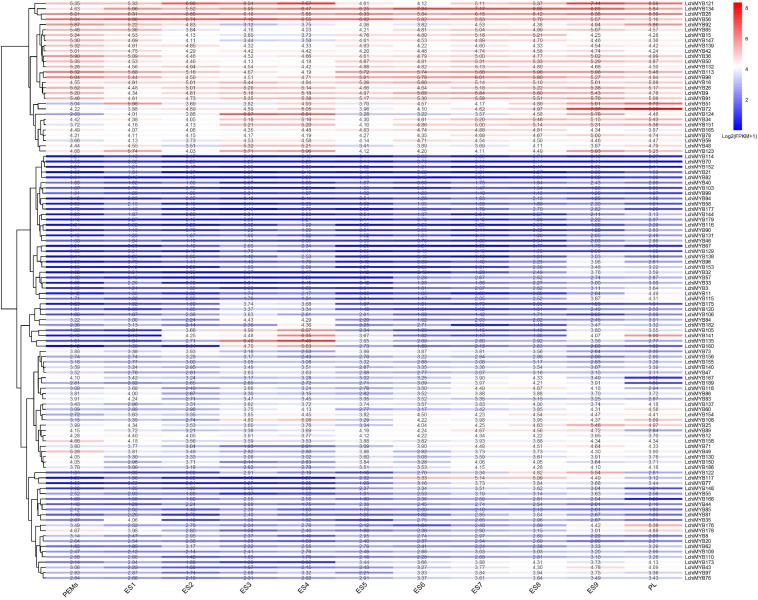
*LchiMYB* gene expression profiles were quantified from RNA-seq data at 11 stages of *Liriodendron* hybrid somatic embryogenesis: proembryogenic mass cultured for 20 days for embryogenic callus (PEMs), then liquid was suspension cultured for 10 days (ES1), single-cell cultured for 2 days (ES2), induced by ABA for 1 day (ES3) and 3 days (ES4), an 7-day globular embryo (ES5), a 13-day heart-shaped embryo (ES6), a 19-day torpedo embryo (ES7), a 25-day immature cotyledon embryo (ES8), a 31-day mature cotyledon embryo (ES9), and 37-day plantlet (PL). The transcript abundance level was normalized and hierarchically clustered, using the log 2 (FPKM + 1) comparison among genes per stage. Colored blocks show decreased (blue) or increased (red) transcript accumulation at different stages. The color legend indicates the fold change expression of *LchiMYBs*, running from low (blue) to high (red) expression. The heatmap was generated, using the R package “pheatmap” (v 1.0.12).

To further explore the time series expression pattern of *LchiMYB* genes, we analyzed their expression during successive stages of somatic embryogenesis (Wang F. X. et al., 2020). In total, 110 (57.89%) *LchiMYB* genes were differentially induced across various stages of somatic embryogenesis (Figure 8). Some *MYB* genes were highly expressed at all stages, while others were less expressed, with only few genes being specifically expressed at certain stages. For example, *LchiMYB121*, –*134*, –*28*, –*56*, *–16*, *–26*, and *–9* were highly expressed at all stages, while *LchiMYB114*, –*70*, –*152*, –*21*, *–82*, *–40*, and *–103* were less expressed at all stages.

At the ES3 and ES4 stages, *LchiMYB135*, *–160*, and –*135* were highly expressed, while showing low expression at other stages. *LchiMYB141* showed high expression at the ES4 and PL stages but low expression at other stages. Notably, some genes had peak expression at a certain stage, such as *LchiMYB51*, –*72*, and –*124* with a peak at the PL, ES9, and ES3 stages, respectively. These results showed that several *LchiMYBs* (R1-MYB and R2R3-MYB) potentially participate in somatic embryogenesis and were differentially expressed at different stages. The four stages imply that ES3, ES4, ES9, and PL may be the main stages regulated by *LchiMYBs*.

### Diversified Expression Patterns of *LchiMYB*s Under Cold, Heat, and Drought Stress

Plants possess different abilities to perceive, respond, and adapt to various biotic and abiotic stressors, which is an important mechanism for plants to survive in adverse environmental conditions (Li J. et al., 2019; Zhou et al., 2019; Pucker et al., 2020). MYB TFs are essential for stress response. However, the mechanism by which *LchiMYB* genes respond to stress is still unknown. In this research, *MYB* gene expression patterns were explored under cold, heat, and drought stress at short intervals (0, 1, 3, 6, and 12 h) and long intervals (1 and 3 days). The TPM value of RNA-seq data from leaf tissues was used to visualize expression trends under different stressors. The TPM value of genes under the three abiotic stressors is shown in Supplementary Table 14.

**FIGURE 9:**
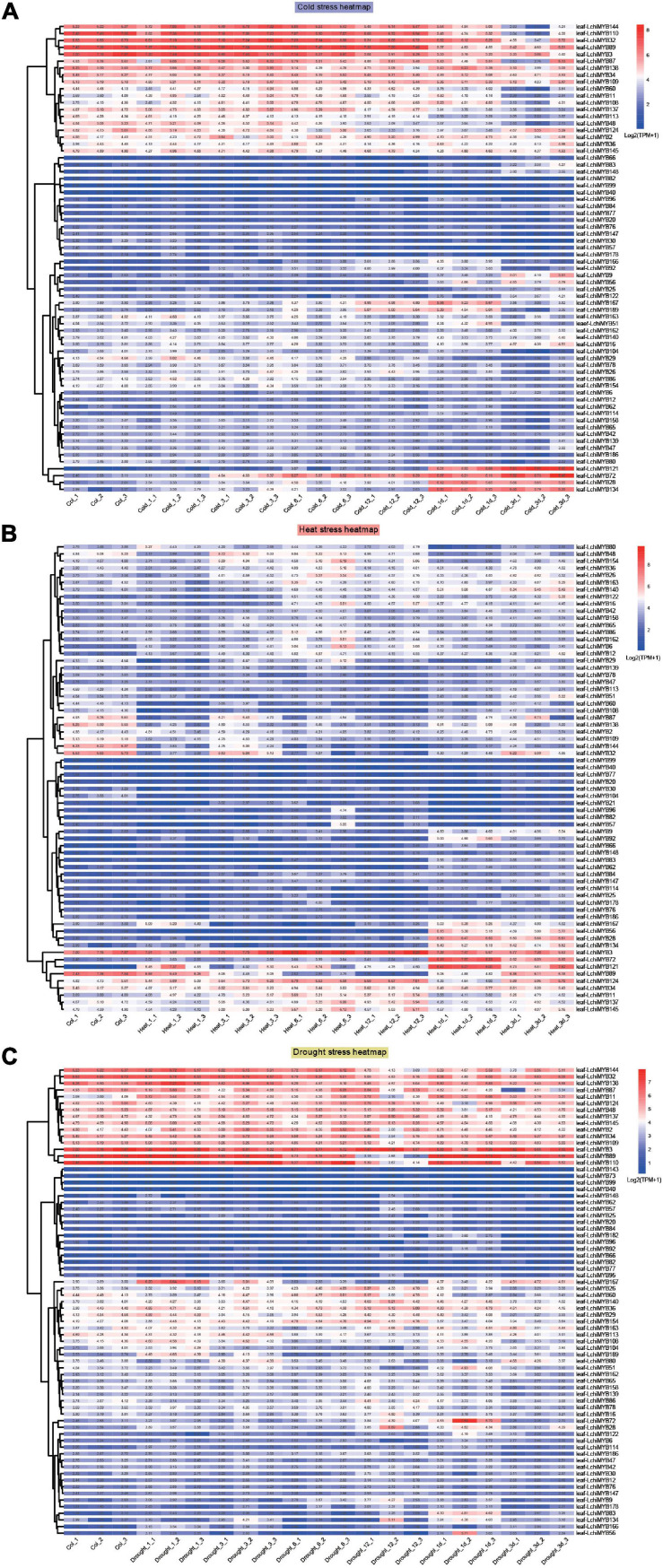
Expression patterns of *LchiMYBs* under cold **(A)**, heat **(B)**, and drought **(C)** stress at six successive time intervals. Ctrl_1, Ctrl_2, and Ctrl_3 represent three biological replicates of the control group; Cold_1, Cold_3, Cold_6, Cold_12, Cold_1d, Cold_3d, Heat_1, Heat_3, Heat_6, Heat_12, Heat_1d, Heat_3d, Drought_1, Drought_3, Drought_6, Drought_12, Drought_1d, and Drought_3d represent three repetitions of each time point (1 h, 3 h, 6 h, 12 h, 1 day, and 3 days); leaf-LchiMYBs represent the leaf tissues used for RNA-seq analysis. The standardization and hierarchical clustering of the transcriptional expression level were calculated by log 2 (TPM + 1) values. Colored blocks show decreased (blue) or increased (red) transcriptional expression among the different time points. The heatmap was generated by the R package “pheatmap” (v 1.0.12).

As shown in Figures 9A–C, under cold stress (Figure 9A), the expression of *LchiMYBs* could be divided into three groups: high expression, low expression at all times, and high expression at long intervals. Under heat stress (Figure 9B), the expression of *LchiMYBs* could be divided into two groups: low expression at all times and high expression at several intervals. Interestingly, under drought stress (Figure 9C), the expression of *LchiMYB*s could also be divided into three groups: high expression, low expression at all times, and high expression at several intervals.

More than 60 *LchiMYB* genes were differentially expressed in response to at least one stressor; most of them were induced by multiple stressors, implying that they play a cross-regulatory role in different signal transduction pathways in response to abiotic stress. For instance, *LchiMYB144*, *–32*, *–89*, and *–3* were induced by all three stressors. *LchiMYB3* was highly expressed under all stressors, while *LchiMYB32* and *LchiMYB144* were only less expressed under heat stress but had high expression under the other two stressors. Similarly, *LchiMYB89* was highly expressed under heat and cold stress but only less expressed under drought stress, suggesting that they are pleiotropic regulators that regulate different environmental condition responses. For instance, *LchiMYB121* responded to cold and heat stress but not to drought stress.

However, some *LchiMYB* genes responded to one stress simultaneously. For instance, three genes (*LchiMYB72*, –*28*, and –*134*) were significantly upregulated by cold treatment at 1– and 3-day intervals; four genes (*LchiMYB145*, –*137*, –*11*, and –*34*) were significantly induced by heat stress; and four genes (*LchiMYB48*, –*137*, –*45*, and –*2*) were significantly induced by drought stress. Additionally, some genes showed opposite expression patterns under different stressors. *LchiMYB134* was significantly upregulated under cold and heat stress but downregulated by drought stress at the 3-d interval. Among these stressors, *LchiMYBs* was more upregulated by long intervals of heat and cold stress than by short intervals, indicating that long intervals were the main biological process of *Lchi*, and *MYBs* play an important role in it.

Combined with the above results, *LchiMYBs* play multiple roles in the response to adversity stress; some genes regulate cold, heat, or drought stress responses, while others regulate the response to two or three stressors. Some genes were not induced by abiotic stress and may be expressed under other types of stress. These results imply that the signaling pathways of plants in response to abiotic stress are complex; long intervals were an important response time interval for *LchiMYBs*. In conclusion, these findings provide valuable information for improving the environmental tolerance of *Lchi*, which can be achieved by genetic manipulation of *LchiMYB* genes.

### qRT-PCR Verification of *Lchi* Under Three Abiotic Stressors

**FIGURE 10:**
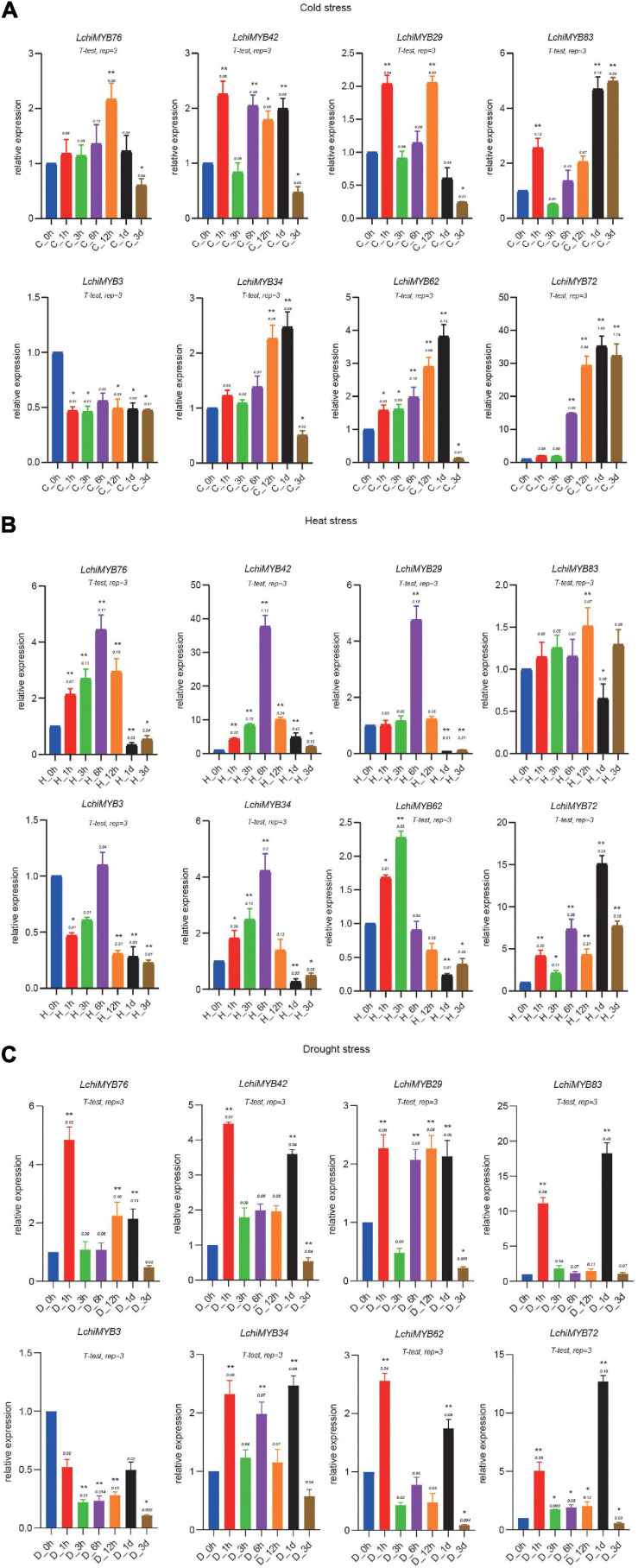
Expression analysis of *LchiMYBs* under cold **(A)**, heat **(B)**, and drought **(C)** stress treatments, analyzed by qRT-PCR. The Y-axis shows the relative expression level, and the X-axis indicates different time points of stress treatment; the mean ± standard error measurement value (SEM) (*n* = 3) is displayed. The *t*-test represents the Student’s test statistic method, and rep = 3 represents the three biological replicates. The group for each treatment time point compared with the control group; the significantly different (*T*-test) *p < 0.05 and **p < 0.01.

qRT-PCR was used to validate the transcriptome data acquired from abiotically stressed leaf tissues of seedlings. Eight genes that were expressed under three abiotic stressors with a different pattern were selected for qRT-PCR experiments (Chen et al., 2017; Liu et al., 2017): two were of the R2R3-MYB type (*LchiMYB83* and *–62*), while the others were of the 1R-MYB type (Figures 10A–C). The qRT-PCR values are presented in Supplementary Table 15. Each *LchiMYB* gene showed a unique expression trend in response to the three abiotic stress conditions.

Under cold stress conditions, all but one *LchiMYB* gene (*LchiMYB3*) showed induced expression. Three genes (*LchiMYB42*, –*29*, and –*83*) showed a 2-fold expression response, as they were strongly upregulated after 1 h; after which, they showed a response again when 6 h to 1 day had passed (Figure 10A). The remaining four genes (*LchiMYB76*, –*34*, –*62*, and –*72*) showed a singular wave of upregulated expression at 12 h and 1 day, with *LchiMYB72* responding very strongly with about 40-fold upregulation and *LchiMYB76*, showing only marginal upregulation (Figure 10A). This showed that 1 h, 12 h, 1 day, and 3 days were the key response intervals, and *LchiMYBs* were upregulated at these time points; perhaps, they have an important regulatory function during these periods.

Under heat stress, five *LchiMYB* genes showed an expression peak point at 6 h, while those of three genes were different, i.e., *LchiMYB83*, *LchiMYB62*, and *LchiMYB72* showed an expression peak point at 12 h, 3 h, and 1 day, respectively (Figure 10B). These genes were induced by heat stress and compared with the control group; there was a peak in expression. Similarly, the *LchiMYB72* showed 15-fold upregulation at the 1-d time point, and *LchiMYB42* showed approximately 40-fold upregulation at the 6-h time point. These results indicate that the 3-h, 6-h, 12-h, and 1-d time points were the main response times for the MYB family under heat stress, and they might play an important role during this period.

Under drought stress, a different, more complex type of expression response was observed than that to temperature stress (Figure 10C). *LchiMYB* genes responded early at 1-h time point, except *LchiMYB3*, which was lower than at 0 h. *LchiMYB42*, –*34*, –*83*, *–72*, and *–62* were highly expressed at the 1-d interval. Some genes, like *LchiMYB29*, were highly expressed at all time points, except at 3 h and 3 days. *LchiMYB76* was highly expressed at the 1-h interval. In contrast to temperature stress, *LchiMYB83* was upregulated by approximately 20-fold, and *LchiMYB72* was upregulated by approximately 12-fold at the 1-d interval. These results suggested that the function of *MYB* is diverse under drought stress, and its signal pathway may be different from that under temperature stress; thus, the regulation mode of *MYB* genes was also different.

In particular, two *MYB* genes (*LchiMYB72* and –*42*) were highly expressed in response to all three tested stressors, suggesting that they function at the intersection of separate biological signaling pathways. In contrast, *LchiMYB3* showed low expression under all three stressors, indicating that it may have no prominent role in stress signaling.

Taken together, our RNA-seq and qPCR data indicated that *LchiMYB* genes were likely involved in the response of *Lchi* to abiotic stress at a particular time, making them important players in the complex signal regulation network and in the adaptation of the tree to various adverse environments.

## Discussion

### *LchiMYBs* Possess Less R2R3-MYBs Genome-Wide and in Highly Conserved Domain Sequences

MYB TFs are the largest family in plants and regulate many gene expressions, which are involved in many biological processes and metabolic pathways (Stracke et al., 2001; Dubos et al., 2010). In *Lchi*, there are no genome-wide reports of the *MYB* gene family and their biological functions, such as growth, development, and abiotic processes. In our study, for the first time, the *MYB* gene family was identified in a series of analyses, including tissue expression, somatic embryogenesis expression, and abiotic stress response.

A total of 190 *MYB* genes were identified, including 76 (40%) R2R3-MYB genes. This result was consistent with the potato *MYB* gene family, which has 111 R2R3-MYBs, accounting for 44% of all 251 *MYB* genes (Li Y. et al., 2020). In many plants, R2R3-MYBs are the largest subgroup in the *MYB* gene family. For instance, *A. thaliana* possesses 126 R2R3-MYBs, which account for 64.29% of all 196 MYBs (Dubos et al., 2010). The *B. distachyon MYB* gene family contains 85 (69.67%) MYB-R2R3s of the total 122 *BdMYBs* (Chen et al., 2017). Pepper (*Capsicum annuum* L.) has 108 R2R3-MYBs, accounting for 50% of all 216 *MYBs* (Wang J. et al., 2020), and *O. sativa* has 109 R2R3-MYBs (58.91%) of the total 185 MYBs (Dubos et al., 2010). These results indicated that R2R3-MYBs were the main subgroup in the plant MYB gene family, which may be associated with the evolutionary status. *Lchi* was considered a basal angiosperm with fewer R2R3-MYBs than higher angiosperms (Chen et al., 2019).

The sequences of the R2R3 domain were analyzed with *A. thaliana*, suggesting that they are highly conservative between different plants. The R2R3 repeats bind upstream of regulatory genes and induce them to express in different tissues or under various environments. Although *Lchi* was a basal angiosperm with an evolutionary status lower than higher angiosperms, such as monocotyledons and dicotyledons (Chen et al., 2019), R2R3-MYB sequences were still conserved in different plants, demonstrating that the function of the *MYB* gene family may be conserved in basal angiosperms (Stracke et al., 2001).

### *LchiMYB* Family Expansion Originated From a Gene Segmental Replication Event

Gene duplication is an important mechanism by which new genes are generated. It contributes significantly to the expansion of the *MYB* gene family in the plant kingdom, leading to the diversification of gene function or driving gene evolution (Yanhui et al., 2006; Dubos et al., 2010). Our results showed that 46 (24.21%) *LchiMYB* genes had segmental repeat events in *Lchi*, which indicated that segmental duplication events were an important driving force for the expansion of *LchiMYB* genes.

Previous studies showed that the *3R-MYB* gene is obtained by replicating one R1 repeat with a 2R-MYB domain, or 3R-MYB is formed by replicating two R2 repeats with a 1R-MYB domain (Millard et al., 2019; Liu et al., 2020; Pucker et al., 2020). For instance, the 1R-MYB gene *LchiMYB73* may be produced by the loss of two R repeats of the 3R-MYB gene *LchiMYB8*. Most gene duplications resulted from segmental duplications, including the R2R3-MYB and 1R-MYB genes. As a result, there are more *MYB* genes in the *Lchi* genome.

Duplicated genes often lose their original function and/or obtain new functions, which increases the adaptability of plants to unfavorable environments. Previous studies showed that the retention of replication genes in the genome leads to diverse expression patterns and responses to various abiotic stressors (Gates et al., 2016; Roy, 2016; Liu et al., 2017; Mmadi et al., 2017). In this study, we found that some gene pairs showed different expression levels under different abiotic stressors. For instance, in one pair, *LchiMYB86* was expressed, while *LchiMYB106* was not expressed under the three tested stress conditions. Further analysis showed that *LchiMYB86* contains certain *cis* elements, such as LTR and MBS elements, which respond to cold and drought stress in its promoter region. In contrast, these elements were not found in the upstream region of *LchiMYB106*.

### *LchiMYB* Genes Regulate the Development of Vegetative and Reproductive Organs

According to previous reports, the potato *StR2R3-MYB* gene has functions in different tissues, such as the leaves, roots, shoots, sepals, stamens, flowers, petals, carpels, and fruit (Li Y. et al., 2020). In *B. distachyon*, *BdMYB055*, *–011*, *–083*, and *–089* were highly expressed in different parts of floral organs (Chen et al., 2017). In *Arabidopsis*, *AtMYB37*, –*38*, and –*84* regulated axillary meristem formation and vegetative processes (Dubos et al., 2010). In this research, eight tissue transcriptomes were evaluated to investigate the function of *LchiMYBs*. *LchiMYBs* are involved in vegetative and reproductive organs, including the bark, phloem, xylem, leaf, bud, sepal, stigma, and stamen. In particular, some *LchiMYBs* were specifically expressed in some tissues; for example, *LchiMYB152* and –*82* were mainly regulated in the phloem, xylem, and stamen but not in other tissues. In the bark and leaves, *LchiMYB118* and –*90* had a high expression pattern, while *LchiMYB70*, –*177*, and –*2* were only expressed in the stamen. These expression patterns indicated that some *LchiMYB* genes were tissue specific and only regulated in their target organs and were not expressed in other tissues. Conversely, some genes were constitutively regulated and had a multi-tissue expression pattern. For example, *LchiMYB124*, –*26*, –*91*, and –*98* were only expressed in the xylem and phloem but not in other tissues.

In summary, *LchiMYBs* have an important function in the vegetative and reproductive organs of *Lchi*, thereby regulating tree growth and flower development. These key *LchiMYB* genes were useful for further exploration of their regulatory function through genetic manipulation and other molecular experiments. In the future, these data will be beneficial to improve the growth, trunk thickening, and reproduction of *Lchi*, thereby increasing the utilization value of wood and reforestation applications.

### *LchiMYB* Genes Have Important Functions at the ABA-Induced Stage in Somatic Embryogenesis

According to a previous report, abscisic acid (ABA), ethylene, and cytokinins (CKs) have important functions during the induction of plant somatic embryogenesis (SE) (Méndez-Hernández et al., 2019). *MYB1R1*-, *MYB98*-, and *MYB26*-like were upregulated in later SE of *Hevea brasiliensis* (Wang et al., 2021). *AtMYB115, AtMYB118*, and *EMK (EMBRYOMAKER)* affected SE in Arabidopsis (Gliwicka et al., 2013). In contrast, ectopically, overexpressing the homeo domain TF WUSCHEL (WUS) and MYB TF *MYB118* formed somatic embryos in *Arabidopsis* (Su Y. H. et al., 2014; Wang F. X. et al., 2020). In *Lchi*, however, *MYB* genes that regulate somatic embryogenesis directly were still not reported. The somatic embryogenesis of the *Liriodendron* hybrid is an important method of asexual propagation to obtain more seedlings. Thus, the *LchiMYB* expression pattern of somatic embryogenesis was explored. Eleven successive stages of somatic embryogenesis were used to obtain RNA-seq data and identify the function of *LchiMYBs*.

Some *LchiMYBs* have shown a powerful regulation function in these 11 stages, such as *LchiMYB121*, –*134*, –*28*, –*56*, *–16*, *–26*, and *–9*. Moreover, after a single cell was cultured for 2 days, it was induced by ABA for 1 day (ES3) and 3 days (ES4), and *LchiMYBs* were highly expressed, including *LchiMYB135*, *–160*, *–141*, and –*135*. At the ES9 and PL stages, *LchiMYB51*, –*72*, and –*124* had peak expression. The expression pattern showed that somatic embryogenesis at specific stages may be regulated by some *MYB* genes, especially at ES3, ES4, ES9, and PL stages during these four periods. Presumably, some *LchiMYBs* were significantly induced by ABA treatment, such as at the ES3 and ES4 stages of somatic embryogenesis, suggesting that ABA-induced *LchiMYBs* regulate the gradual formation of somatic embryos.

Taken together, according to previous reports and the expression pattern, *MYB*s have some vital functions during somatic embryogenesis after being induced by ABA. These results shed a new light on the *MYB* genes involved in the somatic embryogenesis process and ABA-dependent expression patterns. Thus, these ABA-induced *LchiMYBs* were worth further investigation by molecular experiments and revealed their regulatory mechanism. In the future, the regulatory genes of these important stages will be helpful to improve the efficiency of asexual reproduction in *Lchi*, thereby improving the natural reproduction ability of *Lchi* and increasing the yield of trees.

### The Cross-Regulatory Role of *LchiMYB* Families in Response to Cold, Heat, and Drought Abiotic Stress

Overexpression of the *MYB* gene enhances adaptability to abiotic stress. For example, in *Arabidopsis*, overexpression of two wheat genes (*TaMYB33*, *TaMYB73*) enhances salt tolerance (Li J. et al., 2019). Similarly, overexpression of *AtMYB44* increased its tolerance to multiple abiotic stressors (Stracke et al., 2001; Katiyar et al., 2012). Overexpressing *GmMYBJ1* enhances tolerance to drought and low-temperature stress, suggesting its potential function to improve abiotic stress tolerance (Su L.-T. et al., 2014). Some MYB TFs respond to abiotic stress by regulating the expression of the target genes. For instance, *TaMYB19-B* changes the expression of stress-related genes to enhance the ability of wheat to overcome adverse conditions. *TaMYB73* participates in the salt stress response by regulating the expression of stress-related genes (Zhang et al., 2018; Li J. et al., 2019).

Many *MYB* genes may have a potential function in abiotic stress. For instance, in rice, cold stress significantly induced the expression of *OsMYB2* and *OsMYB511*; osmotic stress and exogenous ABA significantly induced the expression of *OsMYB511* and *CMYB1* (Yanhui et al., 2006; Katiyar et al., 2012). In *Arabidopsis*, jasmonate treatment significantly induced the expression of *MYB21/At3g27810*, and *MYB24/At5g40350* (Kranz et al., 1998). Additionally, 150 *MYB* genes were identified in soybeans, 43 of which were significantly upregulated under cold, salt, and drought stress (Baillo et al., 2019; Li J. et al., 2019). Under dehydration, salt, cold, and ABA treatments, *PtsrMYB* was significantly upregulated, which was highly consistent with *AtMYB109* (Sun et al., 2014).

In the present study, we used RNA-seq of heat, cold, and drought stress to explore the functions of *LchiMYBs*, which helps to better understand the cross-regulatory function of *MYB* under multiple stressors. Among these stressors, different expression patterns were observed. *LchiMYB* expression patterns were divided into three groups under cold and drought stress but into two groups under heat stress. Some *LchiMYBs* were expressed under three stress conditions, while others were expressed only under one or two stress conditions. For instance, *LchiMYB3* was highly expressed under all stresses, while *LchiMYB144*, *–32*, and *–89* were induced under the three stressors with different expression patterns. *LchiMYB32*, *–144*, and *–89* were highly expressed under two stressors but less expressed under one stressor. Additionally, *LchiMYB121* was only induced under cold and heat stress. Therefore, these genes were the crucial regulatory factors of *Lchi* under abiotic stress. *LchiMYB3* may be the main regulator, while other genes are secondary regulators, forming a complex abiotic stress signal network of cross-regulation, and the corresponding biological pathways need to be further studied.

Our qRT-PCR results showed that eight selected *LchiMYB* genes with different expression patterns were regulated by cold, heat, and drought stress. The expression of six *LchiMYB* genes was upregulated in response to all three stress conditions. A previous study demonstrated that MBS and LTR are major *cis* elements regulating drought and cold-responsive gene expression (Katiyar et al., 2012; Wang J. et al., 2020).

Most of the *LchiMYB* genes possess these elements. These results further imply that *LchiMYB* genes have a potential cross-regulatory function under abiotic stress.

There were few studies on the abiotic stress resistance of *Lchi*, especially in the whole genome. Because *Lchi* is mainly distributed to the south of the Yangtze River (Chen et al., 2019), these results provide a theoretical basis for the large-scale promotion of *Lchi*, which can be extended to the colder northern region, the higher temperature southern region, and the arid western region of China, and contribute to reducing the greenhouse effect and improving the ecological environment in the future.

## Conclusion

Here, 190 *MYB* members were identified in the *Lchi* genome. These *LchiMYBs* were phylogenetically divided into three major groups, and the R2R3-MYB group (76) was classified into 20 subgroups. The protein sequence features, gene exon-intron structure characteristics, chromosomal locations, phylogeny, and the intra- and inter-species collinearity of the *LchiMYBs* were additionally analyzed. In the evolution process, the MYB family mainly experienced positive selection. The transcriptome of vegetative and reproductive organs showed that *LchiMYB118* and –*90* mainly regulated the development of vegetative organs, and *LchiMYB70*, –*177*, and –*2* mainly regulated the development of reproductive organs. Global gene expression patterns of the 11 stages of somatic embryogenesis suggested that they probably have important functions in the regulation of different *Lchi* somatic embryogenesis stages, especially in the regulation of the ES3, ES4, ES9, and PL stages. Some *MYB* genes may cross-regulate *Lchi* under abiotic stress, such as *LchiMYB3*, *–144*, *–32*, and *–89*. Overall, this study offers further insight into the functional roles of *LchiMYBs*, indicating their regulatory function in growth, development, somatic embryogenesis, and abiotic stress. These results will be helpful to improve the growth and reproduction of *Lchi* by providing a strong theoretical basis for the functional verification of a single gene in the later stage, asexual propagation, and afforestation in different areas.

## Data Availability Statement

The original contributions presented in the study are publicly available. This data can be found here: NCBI, accession numbers: PRJNA679089 and PRJNA679101.

## Author Contributions

WW completed the analysis of *LchiMYBs* and qPCR experiments and wrote the manuscript. SZ performed the analysis. LZ, DW, SL, YLi, and YLu designed and performed the qPCR experiments. JZ provided the transcriptome data for somatic embryogenesis. ZH, TC, JS, and JC designed the project, performed the transcriptome data analysis, and wrote the manuscript. All authors agreed on the whole manuscript.

## Conflict of Interest

The authors declare that the research was conducted in the absence of any commercial or financial relationships that could be construed as a potential conflict of interest.

## Publisher’s Note

All claims expressed in this article are solely those of the authors and do not necessarily represent those of their affiliated organizations, or those of the publisher, the editors and the reviewers. Any product that may be evaluated in this article, or claim that may be made by its manufacturer, is not guaranteed or endorsed by the publisher.

## Footnotes

1 http://www.arabidopsis.org/

2 http://pfam.xfam.org

3 https://www.ncbi.nlm.nih.gov/Structure/cdd/wrpsb.cgi

4 http://gsds.gao-lab.org/index.php

5 https://cran.r-project.org/web/packages/circlize/

6 https://pypi.org/project/jcvi/

7 http://bioinformatics.psb.ugent.be/webtools/plantcare/html/

8 https://www.expasy.org/resources/compute-pi-mw

9 http://www.bioconductor.org/packages/release/bioc/html/ggtree.html

10 https://www.yeastgenome.org/primer3

## Supplementary Material

Supplementary Figure 1 A quantitative real-time PCR (qRT-PCR) dissolution curve and a standard curve of eight genes. The primer specificity and amplification efficiency of eight genes were checked by melting curve analysis and standard curve analysis, respectively. LchiMYBs represent the genes, Lchi18S represents the internal reference gene, and red curves represent the melting and the standard.

Supplementary Figure 2 The Bayesian tree indicating the phylogenetic relationships between 190 Lchi MYB proteins. The proteins are divided into three subgroups, each designated with a different color (e.g., green represents 1R-MYB). The bootstrap values were supported by 1,000 replications and are shown beside the branches.

Supplementary Figure 3 The Bayesian tree indicating the phylogenetic relationships between 76 MYB proteins from *Liriodendron chinense* (*Lchi*) and 126 from *Arabidopsis*. The proteins are divided into 24 main groups, each labeled with a subgroup number (e.g., S1). The bootstrap values were supported by 1,000 replications and are shown beside the branches.

Supplementary Table 1 Fragments per kilobase of the exon model per million mapped fragments (FPKM) value of somatic embryogenesis.

Supplementary Table 2 FPKM value of various tissues for *LchiMYBs*.

Supplementary Table 3 Transcripts per million (TPM) values of mapped *LchiMYBs*.

Supplementary Table 4 Primer sequences of eight *LchiMYBs*.

Supplementary Table 5 Protein size features of *LchiMYBs*.

Supplementary Table 6 Chromosome length of *LchiMYBs*.

Supplementary Table 7 Gene position of *LchiMYBs*.

Supplementary Table 8 Synteny gene pairs of *LchiMYBs*.

Supplementary Table 9 Selective pressure analysis of *LchiMYB*-duplicated gene pairs.

Supplementary Table 10 Protein sequences of 190 *LchiMYBs*.

Supplementary Table 11 Protein sequences of *Lchi* and *ATG* R2R3-MYBs.

Supplementary Table 12 Number and length of the exon-intron structure of *LchiR2R3-MYBs*.

Supplementary Table 13 *Cis* elements of *LchiR2R3-MYBs*.

Supplementary Table 14 TPM value of *LchiMYBs* under cold, heat, and drought stress.

Supplementary Table 15 Quantitative real-time PCR (qRT) value of *LchiMYBs* under cold, heat, and drought stress.

## Abbreviations

*Lchi*: *Liriodendron chinense*
MYB: *Myeloblastosis*
AtMYB: *A. thaliana* MYB
LchiMYB: *Liriodendron chinense* MYB
qRT-PCR: quantitative real-time PCR
MW: molecular weight
aa: amino acid
TPM: transcripts per million
pI: isoelectric point
ATG: *A. thaliana*
MSA: multiple sequence alignment
TFs: transcription factors
HTH: helix-turn-helix
ABA: abscisic acid
FPKM: fragments per kilobase of the exon model per million mapped fragments.
