# Supplementary figures and images for: Characterization of the Liriodendron Chinense MYB Gene Family and Its Role in Abiotic Stress Response
Source: Front Plant Sci. 2021 Jul 26;12:641280. doi: 10.3389/fpls.2021.641280 (PMC8350534; doi:10.3389/fpls.2021.641280)

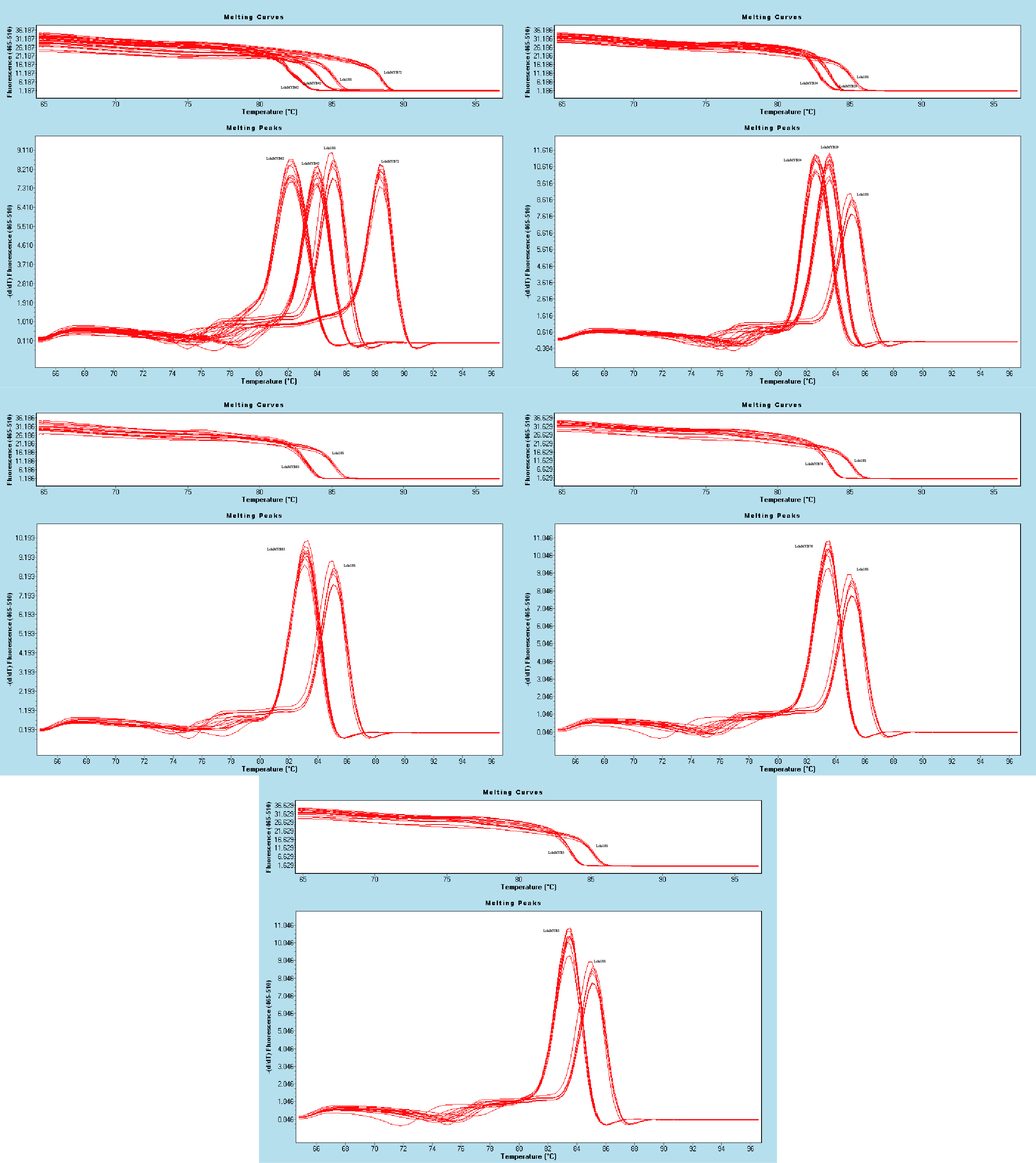

Supplement: Supplementary Figure 1 — A quantitative real-time PCR (qRT-PCR) dissolution curve and a standard curve of eight genes. The primer specificity and amplification efficiency of eight genes were checked by melting curve analysis and standard curve analysis, respectively. LchiMYBs represent the genes, Lchi18S represents the internal reference gene, and red curves represent the melting and the standard. [file Data_Sheet_1.zip › Fig.S1 qRT curve of 8 LchiMYBs.jpg]

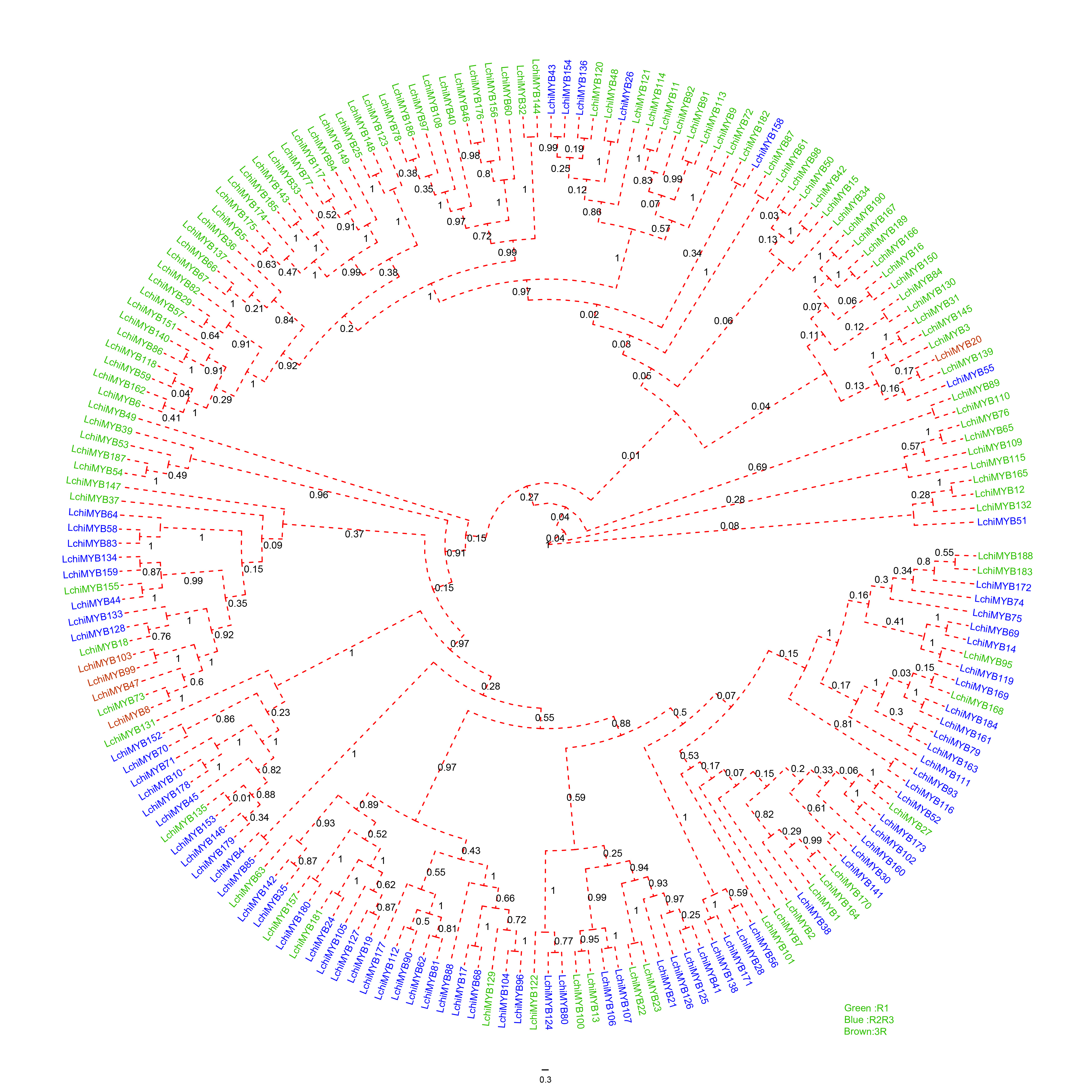

Supplement: Supplementary Figure 1 — A quantitative real-time PCR (qRT-PCR) dissolution curve and a standard curve of eight genes. The primer specificity and amplification efficiency of eight genes were checked by melting curve analysis and standard curve analysis, respectively. LchiMYBs represent the genes, Lchi18S represents the internal reference gene, and red curves represent the melting and the standard. [file Data_Sheet_1.zip › Fig.S2 The Bayes Phylogenetic tree of 190 LchiMYBs.jpg]

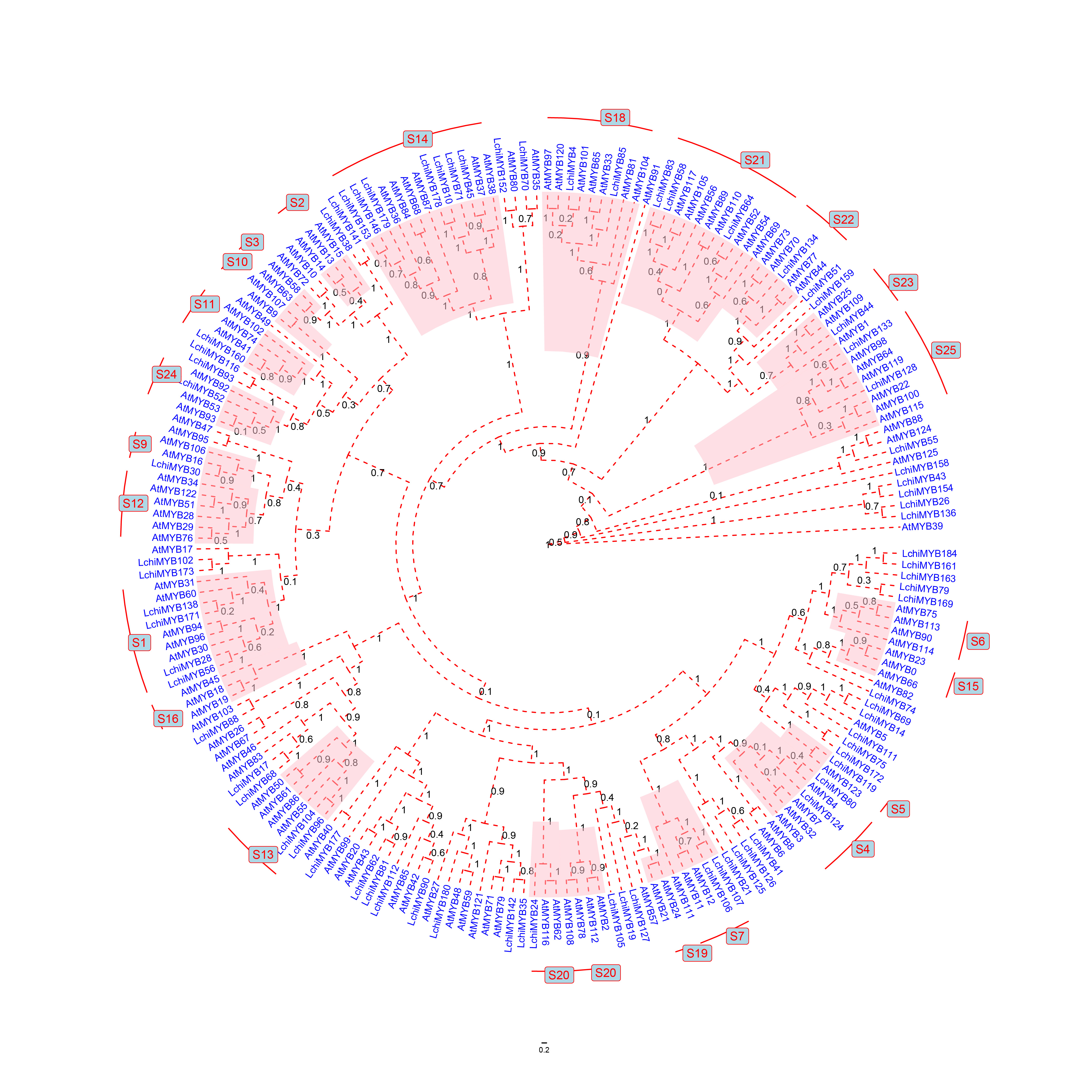

Supplement: Supplementary Figure 1 — A quantitative real-time PCR (qRT-PCR) dissolution curve and a standard curve of eight genes. The primer specificity and amplification efficiency of eight genes were checked by melting curve analysis and standard curve analysis, respectively. LchiMYBs represent the genes, Lchi18S represents the internal reference gene, and red curves represent the melting and the standard. [file Data_Sheet_1.zip › Fig.S3 The Bayes Phylogenetic tree of Lchi and ATG R2R3-MYBs.jpg]
